# Comparative Study of Essential Oils Extracted From *Foeniculum vulgare* Miller Seeds Using Hydrodistillation, Steam Distillation, and Superheated Steam Distillation

**DOI:** 10.1002/fsn3.4593

**Published:** 2024-11-13

**Authors:** Muhammad Haseeb Raza, Muhammad Adnan Ayub, Muhammad Zubair, Amjad Hussain, Samreen Saleem, Muhammad Tauseef Azam, Muzzamal Hussain, Anjuman Gul Memon, Mohamed A. Abdelgawad, Mohammed M. Ghoneim, Ahmed H. El‐Ghorab, Ehab M. Mostafa, Entessar Al Jbawi

**Affiliations:** ^1^ Department of Chemistry University of Sahiwal Sahiwal Pakistan; ^2^ Key Laboratory of Synthetic and Self‐Assembly Chemistry for Functional Organic Molecules Shanghai Institute of Organic Chemistry, Chinese Academy of Science Shanghai China; ^3^ Department of Chemistry, Faculty of Science University of Gujrat Gujrat Pakistan; ^4^ Department of Chemistry University of Okara Okara Pakistan; ^5^ Department of Nutrition and Lifestyle Medicine Health Services Academy Islamabad Islamabad Pakistan; ^6^ Institute of Food and Nutritional Sciences PMAS‐Arid Agriculture University Rawalpindi Pakistan; ^7^ Department of Food Science Government College University Faisalabad Faisalabad Pakistan; ^8^ Department of Biochemistry, College of Medicine Qassim University Buraydah Saudi Arabia; ^9^ Department of Pharmaceutical Chemistry, College of Pharmacy Jouf University Sakaka, Aljouf Saudi Arabia; ^10^ Department of Pharmacy Practice, College of Pharmacy AlMaarefa University Ad Diriyah, Riyadh Saudi Arabia; ^11^ Department of Chemistry, College of Science Jouf University Sakaka Saudi Arabia; ^12^ Department of Pharmacognosy, College of Pharmacy Jouf University Sakaka Saudi Arabia; ^13^ Pharmacognosy and Medicinal Plants Department, Faculty of Pharmacy (Boys) Al‐Azhar University Cairo Egypt; ^14^ Agricultural Extension Directorate, MAAR Damascus Syria

**Keywords:** antimicrobial activity, antioxidant activity, essential oil, *Foeniculum vulgare* Miller, GC–MS, superheated steam distillation

## Abstract

*Foeniculum vulgare* Miller is a highly valued aromatic and nutritious plant. The unique compositions of its essential oil make it more valuable in the flavor, fragrance, and medicinal industries. However, the potential of superheated steam distillation for obtaining essential oils from its seeds has not been explored in detail. This study assessed the composition, yield, antimicrobial, and antioxidant activities of essential oils distilled from *F. vulgare* seeds using traditional hydrodistillation, steam distillation, and superheated steam distillation. Superheated steam distillation resulted in the maximum quantity of essential oil (5.24%) compared to steam (3.47%) and hydrodistillation (2.47%). *Trans*‐anethole, fenchone, estragole, and limonene were the main identified by GC–MS analysis in the essential oils, and these compounds were abundant in the essential oil produced by superheated steam distillation. Essential oil distilled by superheated steam distillation presented the highest antibacterial activity against *Staphylococcus aureus, Pastrulla multocida*, *Bacillus subtilis,* and *Escherichia coli.* The highest antifungal activity against *Aspergillus niger, Fusarium solani, Aspergillus flavus*, and *Alternaria alternate* was also demonstrated by the same essential oil. These findings demonstrated the potential of superheated steam distillation as a highly effective and efficient technique for distilling high‐quality essential oils from *Foeniculum vulgare* Miller seeds. It is suitable for various applications in the food, cosmetic, and pharmaceutical industries.

## Graphical abstract

This study assessed the chemical composition, yield, antibacterial, antifungal, and antioxidant activities of essential oils distilled from *F. vulgare* seeds using traditional hydrodistillation, steam distillation, and superheated steam distillation. Superheated steam distillation resulted in the maximum quantity of essential oil (5.24%) compared to steam (3.47%) and hydrodistillation (2.47%). Trans‐anethole, fenchone, estragole, and limonene were the main identified by GC–MS analysis in the essential oils, and these compounds were abundant in the essential oil produced by superheated steam distillation. Essential oils presented the highest antibacterial activity against *Staphylococcus aureus* , *Pastrulla multocida*, *Bacillus subtilis* , and *Escherichia coli* . The highest antifungal activity against Aspergillus niger, Fusarium solani, Aspergillus flavus, and Alternaria alternate was also demonstrated by the same essential oil. These findings demonstrate the potential of superheated steam distillation as a highly effective and efficient technique for distilling high‐quality essential oils from *Foeniculum vulgare* Miller seeds. It is suitable for various applications in the food, cosmetic, and pharmaceutical industries.

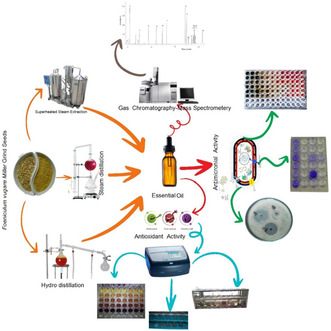

## 1 Introduction

Public health issues related to foodborne illnesses affect millions of individuals each year (Ravindran, Subha, and Ilangovan 2020). Pathogenic microorganisms, *Staphylococcus aureus, Listeria monocytogenes, Klebsiella pneumoniae, Escherichia coli*, and *Campylobacter jejuni* , are commonly responsible for causing a range of infections (Natta et al. 2008). Tert‐butylhydroquinone (TBHQ), butylated hydroxyanisole (BHA), and butylated hydroxytoluene (BHT) are synthetic antioxidants commonly utilized in the food industry to prolong shelf life by microbial growth. However, these compounds may have negative consequences on human health, including liver dysfunction, DNA damage, and premature aging (Himani et al. 2022). Despite these potential health risks, various food industries continue to employ these synthetic antioxidants to enhance shelf life (Saǧdıç and Özcan 2003).

Recent research has shown that phytoextracts have the potential to be an effective and natural alternative to synthetic preservatives owing to their antioxidant and antimicrobial properties (Jabeen et al. 2023). Essential oils (EOs) are concentrated phytoextracts used in aromatic medicine, paints, cosmetics, and perfumes (Mertens, Buettner, and Kirchhoff 2009; Mothana et al. 2011). EOs offer a wide range of potential health benefits, with their ability to prevent oxidative stress and related diseases being a major focus of research (Miguel 2010). The growing interest in natural and plant‐derived sources has driven renewed attention to EOs. As a result, EOs have gained significant interest as an alternative to synthetic antioxidants and are increasingly being investigated for their potential therapeutic benefits. Similarly, *F. vulgare* EO has been reported to have several biological activities, including hepatoprotective, anti‐diabetic, anti‐thrombotic, anti‐inflammatory, anti‐cancer, anti‐fungal, and anti‐bacterial activities (Badgujar, Patel, and Bandivdekar 2014).

There are various techniques for extracting essential oils, such as steam distillation (SD), hydrodistillation (HD), subcritical water extraction (SWE), supercritical CO_2_ extraction (SCOE), and superheated steam distillation (SHSD). HD and SD are two conventional methods that employ heat and steam to extract essential oils from plant biomass. Both methods are effective, but steam distillation may be slightly more efficient. However, these methods can decompose labile compounds, consume more fuel, and take longer to extract essential oils (Mothana et al. 2011).

The use of superheated steam distillation (SHSD) has recently gained popularity as a preferred method for extracting essential oils from plants and herbs due to its potential advantages over subcritical water extraction, supercritical fluid extraction, and other conventional methods (Ayub et al. 2022). SHSD is an extraction method that utilizes steam at temperatures exceeding the boiling point of water. Compared to conventional distillation techniques, SHSD is more efficient, allowing for a more direct transfer of heat to the plant material, which results in faster and more complete extraction of EOs. Moreover, research has demonstrated that SHSD significantly increases the yield of essential oils from thyme (Rouatbi, Duquenoy, and Giampaoli 2007), *Boswellia serrata* oleo‐gum‐resin (Ayub et al. 2022), *Syzygium aromaticum* L. (Ayub, Choobkar, et al. 2023), and *P. roxburghii oleoresin* (Ayub, Goksen, et al. 2023).

Fennel, *Foeniculum vulgare* ( *F. vulgare* ), belongs to the Apiaceae family and has a fragrant aroma. It has long been used in the flavor and medicine industries. *F. vulgare* is quite effective in managing a range of infectious diseases caused by bacteria, parasites, viruses, mycobacteria, and protozoa (Dua, Garg, and Mahajan 2013; Kaur and Arora 2009). The most valuable compounds in *F. vulgare* include fenchone, trans‐anethole, methyl‐chavicol, and alpha‐pinene. Fenchone is used as an anti‐irritant; there are several uses for limonene, including solvents, gums, wetting agents, and scattering agents. In perfumery, anethole enhances scents in cosmetics, soaps, and pharmaceutical products, while methyl‐chavicol, or estragole, is an important component of perfume and flavor in the food and liquor industries. α‐Pinene is used in the production of camphor, bug sprays, solvents, and perfume bases (Rather et al. 2016).

Despite the therapeutic potential of this EO, limited studies have explored the percentage yield, antioxidant properties, and antimicrobial activity of *Foeniculum vulgare* Miller seeds EO obtained through hydrodistillation, steam distillation, and superheated steam distillation. The present study aims to fill this research gap by comparing these three extraction techniques for *Foeniculum vulgare* Miller EO.

## 2 Materials and Methodology

### 2.1 *F.F.* Extraction Methods

#### 2.1.1 Hydrodistillation (HD)

EO of *F. vulgare* Miller seeds was obtained by employing a Clevenger‐type device comprising of a flask, heating mantle, condenser, and a separating funnel. To isolate the EO, 300 g of *F. vulgare* Miller seeds with a mesh size of 80 were mixed with 3 L of distilled water in the flask and subjected to heat using an electrical heating mantle for a duration of 3 h. Subsequently, the resulting hydrosol and EO were collected in a separating funnel. The EO was separated from the hydrosol through decantation, followed by removal of any residual moisture using 0.25–0.50 g of anhydrous Na_2_SO_4_. Finally, the EO was stored in opaque black glass bottles for further analytical evaluation (Ayub et al. 2018).

#### 2.1.2 Steam Distillation (SD)

The steam distillation process was employed to extract essential oil (EO) using a range of apparatus, including a Clevenger‐type apparatus and biomass flask. The round bottom flask contained water, which was heated using electricity, generating steam. Seeds of *F. vulgare* Miller weighing 100 g were placed in a cotton bag and inserted into the flask, which was vertically positioned on top of the Clevenger type. Steam permeated the glandular trichomes of the *F. vulgare* Miller seeds and extracted the EO, which was separated from the hydrosol through a separating funnel. Anhydrous Na_2_SO_4_ weighing 0.25–0.50 g was introduced to remove moisture from the EO, which was then filtered and stored in black glass vials for subsequent analysis (Azeem et al. 2021).

#### 2.1.3 Superheated Steam Distillation (SHSD)

Superheated steam distillation equipment (SHSD‐001, PAMICO Technologies, Faisalabad, Punjab, Pakistan) was utilized to extract the EO from the seeds of *F. vulgare* Miller. The equipment comprises several components, including a S.S superheated steam generator, S.S biomass extraction chamber, S.S condensers and chiller, and a glass hydrosol collection vessel. The extraction process involved the use of 300 g of plant biomass in the extraction chamber, and steam supply lines were equipped with thermocouples that monitored the steam temperature. The steam was generated at a pressure of 75 psi and a temperature of 150°C. Following a 60‐min exposure to superheated steam, 0.25–0.50 g of anhydrous Na_2_SO_4_ was added to the extracted essential oils to remove any moisture. A microfilter was used to filter the oil, and amber glass vials were utilized for further analysis. To ensure the reliability of the findings, the extraction process was repeated five times. In summary, this state‐of‐the‐art distillation equipment effectively extracted essential oils from *F. vulgare* Miller seeds (Ayub et al. 2022).

### 2.2 Antioxidant Assays

#### 2.2.1 DPPH (1,1‐Diphenyl‐2‐Picrylhydrazyl) Free Radical Scavenging Activity (DPPH‐FRSA)

In order to assess the efficacy of essential oils (EO) as a means of scavenging free radicals, a modified DPPH‐FRSA was conducted according to the established protocols (Mensor et al. 2001). The protocol involved combining 1 mL of a 0.3 M DPPH solution with various concentrations of essential oils, which were then left to incubate in darkness for a period of 20 min. Following this, the absorbance of both samples and the control was recorded at 518 nm. The control sample was made by mixing 1 mL ethanol with 2.5 mL of DPPH, without EO and gallic acid standard, while the positive control was made by blending 2.5 mL of only gallic acid standard with 1 mL of DPPH solution. The DPPH scavenging activity was considered as a percentage by the following equation:

$$\text{DPPH scavenging activity}\;\left(\%\right)=\left(\frac{\mathrm{Abs}\;\text{control}-\mathrm{Abs}\;\text{sample}}{\mathrm{Abs}\;\text{control}}\right)\times 100$$

#### 2.2.2 Hydrogen Peroxide (H_2_O_2_ ) Scavenging Activity

H_2_O_2_ scavenging activity of all EOs was investigated using spectrophotometric analysis based on established protocols (Asadim 2008). To carry out the analysis, a phosphate buffer with a pH of 7.4 and a concentration of 0.17 M was prepared, and a 2 mM H_2_O_2_ solution. was added. The prepared hydrogen peroxide solution was mixed with 1 mL of each EO distilled by each distillation method. The obtained product was incubated for 10 min at 25°C. Using a UV‐spectrophotometer with a blank, absorbance at 230 nm was recorded for the samples after treatment. This same procedure was also applied for the samples treated with ascorbic acid (100 ppm). The scavenging activity of H_2_O_2_ in the samples was measured using the following formula:

$$\text{Hydrogen Peroxide scavenging activity}\;\left(\%\right)=\left(\frac{A_{0}-A1}{A_{0}}\right)\times 100$$

Here, *A*_0_ = absorbance of the control, *A*_1_ = absorbance of the sample.

#### 2.2.3 Total Antioxidant Contents (FRAP Assay)

The total antioxidant contents of EO were evaluated by following the FRAP assay with minor modifications (Habila et al. 2010). This method was employed, involving the addition of 1 mL of each oil to a solution of 2.3 mL of 2.5 mL of 1% w/v potassium ferricyanide and pH 6.6 phosphate buffer (0.2 M). This was followed by incubation at 37°C in a water bath for 20 min. Next, 2.5 mL of a 10% w/v trichloroacetic acid solution was added, and the mixtures were subjected to centrifugation at 1000 rpm for 10 min. Subsequently, 2.5 mL from every sample solution was blended with 0.5 mL of 0.1% w/v FeCl_3_, diluted with 2.5 mL of distilled water, and treated with 0.5 mL of ferric chloride solution (0.1% w/v). The absorbance of each sample was measured at 700 nm using a UV–Vis spectrophotometer. A calibration curve was drawn using gallic acid as the standard, and the total antioxidant content was expressed as mg/mL of GAE.

### 2.3 Antimicrobial Activity

#### 2.3.1 Disk Diffusion Method

The antifungal and antibacterial activity of *F. vulgare* Miller seeds EOs were assessed by the disk diffusion technique. To gauge the antibacterial potential of *F. vulgare* Miller seeds EO, *Bacillus subtilis‐*ATCC 10707, *Staphylococcus aureus‐*ATCC 25923, *Pastrulla multocida‐*ATCC 43137, and *Escherichia coli‐*ATCC 25922 were selected as bacterial strains. Similarly, the antifungal potential of essential oil was assessed against *Fusarium solani‐*ATCC 36031, *Aspergillus niger*‐ATCC 10575, *Alternaria alternate‐*ATCC 20084, and *Aspergillus flavus‐*ATCC 20046. The Department of Microbiology at the UAF, Pakistan validated the authenticity and purity of the strains. Nutrient agar‐NA and potato dextrose agar‐PDA were utilized to culture bacterial and fungal strains, respectively. The bacterial culture was maintained in incubation at 37°C for 24 h, while the fungal culture was kept in incubation at 30°C for 48 h. A 100 μL suspension of bacteria (1 × 10^8^ CFU/mL) inoculated on NA and fungi (1 × 10^4^ spores/mL) on PDA. Furthermore, 15 μL of essential oil solutions (1 mg/mL of DMSO) from each technique were placed on 6 mm‐sized paper disks. To compare the sensitivity of bacteria and fungi, amoxicillin and fluconazole (30 μg/dish) were employed as positive controls, while disks without samples were utilized as negative controls. Antimicrobial activity of EO against microbes was determined by calculating the inhibition zone in millimeters (Kiehlbauch et al. 2000).

#### 2.3.2 Resazurin Microtiter Plate Assay (RM‐Plate Assay)

The RM‐plate test was utilized to evaluate the MIC of EOs against bacterial strains. In order to maintain sterility, polymeric microtiter 96‐well plates were sterilized, primed, and labeled in a sterile environment. EOs (100 μL) were dispersed in DMSO (10% V/V) and arranged in the first row of the plate, while M‐H broth (50 μL) was introduced into all the remaining wells. The EOs were diluted in decreasing concentrations (10–0.046 mg/mL) using M–H broth. After dilution, each well contained 50 μL of the sample, 10 μL of resazurin indicator solution, and 10 μL of each microbial solution. To maintain the microbes' hydration, each well was delicately wrapped with cling film. Resazurin solution was produced by dissolving 270 mg tablet in 40 mL distilled water. To ensure the solution was homogenous and entirely dissolved, a vortex mixer was utilized. Each plate had a column that served as the positive control, which was standard antibiotics, a negative control consisting of all the solutions without antimicrobial samples, and a column containing antimicrobial samples with 10 μL of M‐H broth. Subsequently, the plates were subjected to an incubation period of 18–24 h at 37°C. During this time, a change in color was observed with a transition from the original purple hue to either a pink or colorless tone, indicating positive shifts. The MIC was estimated by assessing the minimum concentration of the sample at which the color shift was detected. The MIC value was obtained by averaging the results for the samples and microbial strains across three experiments (Sarker, Nahar, and Kumarasamy 2007).

#### 2.3.3 Micro‐Dilution Broth Susceptibility Assay (MDBS Assay)

The MDBS test was utilized to assess the MIC of EOs against fungal strains. EOs were first dissolved in 10% DMSO before being utilized in culture media. A microtiter plate consisting of 96 wells was utilized to assess the efficacy of EOs in growth control, sterility control, and solvent control. Dilutions of EOs, with concentrations from 0.01 mg/mL to 30.0 mg/mL, were prepared and added to the wells. To commence the assay, microplates were prepared by adding 20 μL of the test solution and 160 μL of Sabouraud‐dextrose‐broth, followed by seeding with 20 μL of a standard microorganism suspension with a concentration of 5 × 10^5^ cfu/mL. The MIC value was calculated by incubation of samples at 37°C for 18–24 h. It was calculated by examining a shift in color from purple to pink or colorless, indicating positive results. Following this, plates were placed in an incubator for 48 h at a temperature of 30°C. Positive control was 1.0 mg/mL of Fluconazole in 10% DMSO. The identification of fungal growth was done by detecting a whitish pellet at the bottom of wells. By determining the highest concentration of the test strains that completely ceased to proliferate, the MIC was then established (Dabur et al. 2004).

#### 2.3.4 Biofilm Inhibition Activity

Biofilm inhibition activity of EOs distilled by different distillation methods was performed against *Bacillus subtilis* (ATCC‐10707) and *Staphylococcus aureus* (ATCC‐25923), Gram‐positive bacteria, and *Pastrulla multocida* (ATCC‐43137) and *Escherichia coli* (ATCC 25922), Gram‐negative bacteria, following protocols defined by Shahid et al. (2015) with minor modifications. Nutrient broth (100 μL), EO sample (100 μL), and bacterial culture (10 μL) were added to each well of the well plate. After incubation, prepared well at 37°C for 18 h, the material was *trans*ferred, the new microbial culture was cleaned twice by PBS (220 μL), and the biofilm was static for 15 min with methanol (99%) and marked for 10 min with crystal violet (7%). The residual strains were washed using deionized water. Glacial acetic acid (33%) was employed as a biofilm solvent. The OD (optical density) of each well was taken at 630 nm in contrast to the negative control, which included only nutrient broth and bacterial culture, and *Rifampicin* as sample replacement in the positive control. The inhibition percentage was measured using the following formula:

$$\%\text{inhibition}=100-\frac{O.D.\;\text{of sample}}{O.D.\;\text{of negative control}}\times 100$$

### 2.4 Gas Chromatography–Mass Spectrometry

Gas Chromatography–Mass Spectrometry was employed to detect the volatile compounds of EOs from each distillation method. A Shimadzu (GC‐2010) system with a mass detector (QP‐2010) was utilized in conjunction with a DB‐5 capillary column (dimensions = 50 m × 0.25 mm) and 0.25 μm film. The injection port was used to inject 1 μL of EO diluted with n‐hexane (1:10) through a syringe. The temperature of the column was gradually raised and kept at 240°C, and N was employed as the carrier gas with 1.5 mL/min flow rate. Mass spectra of the volatile compounds were obtained using electron ionization mode (70 eV). Retention indices (RIs) of the compounds identified in the study and n‐alkanes (C9–C24) standards were utilized. The RIs obtained, along with the mass spectrometry (MS) data, were then compared to the NIST library for analysis. The quantification of components from essential oil followed the methodology described in the literature, with undecane used as an internal standard to compute response factors. Traditional standards were injected to confirm several compounds (Ayub et al. 2018).

$$\mathrm{RFc}=\frac{\mathrm{Ac}/\mathrm{Ais}\;}{\mathrm{Cc}/\mathrm{Cis}}$$

RFc represents the EO component's response factor, Ac and Ais are the EO component's and internal quality's peak regions, and Cc and Cis concentrations are also linked. R.F.s of 1.0 were assigned to minor, unknown peaks.

$$\begin{array}{c} \text{Response Factor For The Same Component}\\ \;=\frac{\text{Peak Area For The Component}}{\text{Response Factor For The Same Component}} \end{array}$$

$$\text{Percentage}\;\left(\%\right)=\frac{\text{corrected area for the component}\;}{\text{total of corrected areas}}\times 100$$

### 2.5 Statistical Analysis

Each experiment was conducted three times, and data were statistically analyzed using STATISTICA 5.5 software and ANOVA with Tukey HSD post hoc test. Statistical significance was determined as a probability value of *p* ≤ 0.05. Data are provided as mean values and standard deviations based on triplicate assays.

## 3 Results and Discussion

### 3.1 Chemical Composition

The chemical composition of *Foeniculum vulgare* ( *F. vulgare* ) essential oils (EOs) extracted by hydrodistillation (HD), steam distillation (SD), and superheated steam distillation (SHSD) was analyzed using GC–MS. Table 1 indicates that the EOs extracted by HD, SD, and SHSD contained 23, 20, and 17 compounds, respectively. The major compounds in all three EOs were trans‐anethole (58.48%–66.46%), followed by fenchone (8.4%–9.8%), estragole (6.23%–7.13%), and limonene (6.5%–7%). *trans*‐Anethole is the most abundant isomer of natural anethol, which is an alkyl phenol ether (Foroughi et al. 2017). Highest concentrations of different bioactive compounds were found in the EO extracted by SHSD, which was primarily composed of oxygenated monoterpenes (76.58%–86.23%), followed by monoterpenes (9.8%–11.06%), sesquiterpenes (0.79%–1.42%), and oxygenated diterpenes (0.14%–0.62%).

**TABLE 1:** The chemical composition of essential oils extracted by steam distillation, hydrodistillation, and superheated steam distillation from *F. vulgare* Miller seeds.

Sr. no	Components	RI*	Hydrodistillation	Steam distillation	Superheated steam distillation	Method of identification
1	α‐Pinene	938	1.27 ± 0.04^c^	1.33 ± 0.01^b^	1.44 ± 0.02^a^	a,b
2	Camphene	950	0.82 ± 0.01^a^	0.16 ± 0.01^c^	0.22 ± 0.01^b^	a,b
3	β‐Pinene	976	0.35 ± 0.01^c^	0.41 ± 0.01^b^	0.54 ± 0.01^a^	a,b
4	α‐Phellandrene	1006	0.12 ± 0.01^c^	0.23 ± 0.01^b^	0.33 ± 0.01^a^	a,b
5	β‐Ocimene	1026	0.06 ± 0.01^c^	0.79 ± 0.01^a^	—	a,b
6	Limenone	1031	6.50 ± 0.02^c^	6.7 ± 0.04^b^	7.00 ± 0.01^a^	a,b
7	3‐Carene	1032	0.34 ± 0.01^c^	0.4 ± 0.02^b^	0.66 ± 0.03^a^	a,b
8	γ‐Terpinene	1055	0.34 ± 0.01^c^	0.66 ± 0.01^b^	0.87 ± 0.02^a^	a,b
9	Fenchone	1090	8.40 ± 0.03^c^	9.20 ± 0.02^b^	9.80 ± 0.04^a^	a,b
10	Camphore	1148	0.62 ± 0.03^a^	0.48 ± 0.04^b^	0.40 ± 0.01^c^	a,b
11	Estragole	1202	6.23 ± 0.03^c^	6.87 ± 0.04^b^	7.13 ± 0.02^a^	a,b
12	Fenchyl acetate	1230	0.86 ± 0.01^a^	—	—	a,b
13	Carvone	1242	0.66 ± 0.01^c^	0.74 ± 0.02^b^	0.81 ± 0.03^a^	a,b
14	Trans‐anethol	1288	58.48 ± 0.03^c^	62.38 ± 0.02^b^	66.46 ± 0.01^a^	a,b
15	Eugenol	1351	0.23 ± 0.01^b^	0.12 ± 0.01^c^	0.34 ± 0.01^a^	a,b
16	α‐Copaene	1378	0.44 ± 0.01^b^	0.55 ± 0.03^a^	—	a,b
17	Carveol	1390	0.07 ± 0.01^b^	0.07 ± 0.01^b^	0.09 ± 0.01^a^	a,b
18	Caryophyllene	1437	0.73 ± 0.01^a^	0.63 ± 0.02^c^	0.64 ± 0.03^b^	a,b
19	β‐Farnesene	1458	0.09 ± 0.01^a^	0.09 ± 0.01^a^	—	a,b
20	Germacrene D	1502	0.49 ± 0.01^c^	0.50 ± 0.02^b^	0.56 ± 0.01^a^	a,b
21	α‐Amorphene	1506	—	—	0.23 ± 0.00^a^	a,b
22	Cinnamaldehyde	1561	0.30 ± 0.01^c^	0.42 ± 0.01^b^	0.56 ± 0.01^a^	a,b
23	β‐Caryophyllene oxide	1581	0.40 ± 0.01^a^	—	—	a,b
24	Phytol	1949	0.14 ± 0.02^b^	0.62 ± 0.01^a^	—	a,b

*Note:* a = Identification based on retention index; b = Identification based on comparison of mass spectra.

* Compound listed in order of elution from a DB‐5 capillary column and retention indices on the DB‐5 capillary column.

The higher concentration of major compounds in the SHSD‐distilled EO in this study may be due to their higher boiling points or greater solubility in steam, which allows them to be more easily vaporized and collected. It has been reported that high temperatures increase the polarity of steam molecules and increase the diffusivity of compounds in steam (Plaza and Marina 2019; Teo et al. 2010). In addition, prolonged exposure to high temperatures can also cause degradation, solubilization, or the formation of unwanted constituents, reducing yield and selectivity (Plaza and Marina 2019; Teo et al. 2010). These results showed the absence of β‐ocimene, fenchyl acetate, β‐caryophyllene oxide, β‐farnesene, α‐copaene, and phytol in the SHSD‐distilled EO. It is possible that these compounds were denatured by the high temperature of superheated steam (Gamiz‐Gracia and De Castro 2000), but it is also important to consider that the concentration of each compound in the distilled EO depends on various factors, including volatility, solubility in steam, and the temperature and pressure of the distillation process.

According to previously published data, *trans*‐anethole is the major component of *F. vulgare* Miller essential oil (EO). The major oxygenated monoterpenes in our study were trans‐anethole, fenchone, and estragole, while limonene dominated. In contrast, sesquiterpenes and oxygenated diterpenes had β‐caryophyllene oxide and phytol as their major compounds, respectively.

According to our findings, *F. vulgare* Miller essential oil (EO) contains a wide range of components. According to Anwar et al. (2009), *trans*‐anethole constituted the majority of the volatile oil extracted from *F. vulgare* seeds by hydrodistillation (HD). Concentrations of trans‐anethole, estragole, fenchone, and limonene were found to be respectively 69.87%, 10.23%, 4.50%, and 5.45%, respectivelly. Diao et al. (2014) also identified trans‐anethole, fenchone, limonene, and estragole as the main components of *F. vulgare* seeds EO. Similarly, Khammassi et al. (2018) determined that trans‐anethole, fenchone, and limonene were the major chemical components of Tunisian *F. vulgare* seeds EO extracted by HD. Pecarski, Dragićević‐Ćurić, and Jugović (2017) found that *F. vulgare* seeds EO extracted by HD contained *trans*‐Anethole (63.13%), followed by fenchone (15.53%), estragole (6.43%), α‐pinene (4.33%), and limonene (4.69%) as main compounds. Miguel (2010) reported that *F. vulgare* aerial parts EO extracted by HD contained *trans*‐Anethole (31%–36%) as a major compound. Gende et al. (2009) found E‐anethole (92.7%) as the major compound in *F. vulgare* EO extracted by HD. Foroughi et al. (2017) reported the highest concentration of trans‐anethol in their findings.

Different chemical compositions of EOs might be caused by the cultivation season, geography, and maturity of plants (Diao et al. 2014). The maximum concentration of the major compounds was observed in the SHSD‐distilled EO, while the minimum concentration was observed in the HD‐distilled EO. This is in good agreement with Sadeghmousavi and dastpak (2019), who found that subcritical water extraction gave a higher anethole concentration (0.627%) than hydrodistillation (0.0936%). In another study, SCOE extracted 14.09% higher concentration of *Trans*‐anethol than simultaneous distillation extraction and also reported the higher concentration of oxygenated monoterpenes in EO extracted by SCOE than the SDE (Díaz‐Maroto et al. 2005). Damjanović et al. (2005) also found that SC‐CO_2_ gave higher concentration of *trans*‐anethol and other oxygenated monoterpenes in *F. vulgare* seeds EO than that of HD.

According to our results, SHSD proves to be the most efficient technique for essential oil extraction from plant biomass at a temperature of 150°C. The SHSD technique was able to extract the highest concentrations of major chemical compounds, demonstrating its ability to selectively extract these compounds. This suggests that SHSD may be a useful technique for selectively isolating specific chemical compounds from *F. vulgare* seeds.

### 3.2 Percentage Yield

**FIGURE 1:**
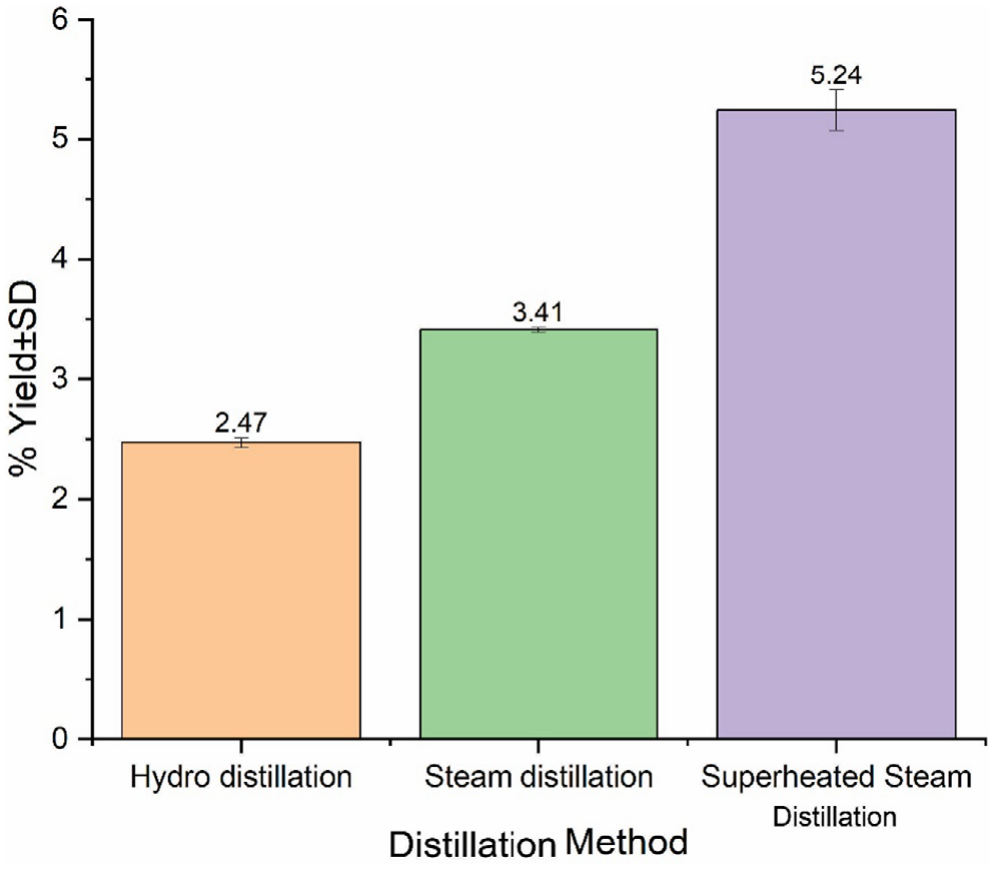
Comparison of essential oil % yield from *F. vulgare* Miller seeds using hydrodistillation, steam distillation, and superheated steam distillation.

The yield of EO extracted from *F. vulgare* seeds was evaluated using three different techniques: superheated steam distillation (SHSD), steam distillation (SD), and hydrodistillation (HD). Current results, depicted in Figure 1, indicate that the percentage yield of EO varied based on the extraction method employed. The SHSD technique, which utilized superheated steam at a temperature of 150°C, yielded 53% and 45% more EO than the HD and SD techniques over a 60‐min period, respectively.

The higher yield of EO obtained through SHSD may be attributed to the enhanced solubilization capacity at higher temperatures compared to the range of 100°C–140°C, as noted by IAPWS (International Association for the Properties of Water and Steam) (Wagner and Kretzschmar 2008). In addition, high temperature creates the high kinetic energy in the superheated steam, enabling it to behave like a gas, allowing it to effectively burst glandular trichomes and increase the evaporation of bioactive compounds through diffusion in oily glands (Cengel 2011).

The polarization, permittivity, viscosity, density, and surface tension of water are also affected by higher temperatures, causing an increase in diffusivity (Plaza and Marina 2019). The mass transfer rate is improved by the increased diffusivity through better wetting of the matrices and penetration through the matrix particles, resulting in improved extraction process efficiency.

The SHSD technique has been demonstrated to effectively extract high yields of EOs from various plant sources. Notably, Rouatbi, Duquenoy, and Giampaoli (2007) successfully distilled significant amounts of thyme EO, while Ayub et al. (2022) reported higher yields of *Boswellia serrata* oleo‐gum‐resin EO. Recently, Ayub, Choobkar, et al. (2023), Ayub, Goksen, et al. (2023) extracted a higher amount of bioactive compounds from *Syzygium aromaticum* L., as well as achieving a higher yield of *P. roxburghii* oleoresin EO. Moreover, our findings on the EO yield of *F. vulgare* Miller seeds align with the results of previous studies that have utilized hydrodistillation (HD) and steam distillation (SD) techniques. Anwar et al. (2009) obtained a 2.81% yield of EO from *F. vulgare* seeds using HD, while Roby et al. (2013) reported a yield of 1.95% using the same method. Similarly, Khorshidi et al. (2009) found a yield of 3.53% using HD on *F. vulgare* seeds. In contrast, Fang et al. (2006) obtained a lower yield of 0.98% using SD, and Zheljazkov et al. (2013) reported a yield of 0.68% using SD on *F. vulgare* fruit.

Additionally, Milenković et al. (2022) extracted EO from the stem and leaves of *F. vulgare* and obtained yields of 0.21% and 0.83%, respectively. Piccaglia and Marotti (2001) reported EO yields ranging from 0.20% to 0.38% from different organs of 13 wild *F. vulgare* plants using the EO technique. Damayanti and Setyawan (2012) obtained a yield of 2.04% from *F. vulgare* seeds using SD, while Mohamad et al. (2011) reported a yield of 1.83% through steam distillation. Benmoussa et al. (2019) found a yield of 0.45% using enhanced solvent‐free microwave extraction, and Dong et al. (2022) obtained a yield of 1.05% using dual‐cooled microwave extraction on *F. vulgare* seeds.

Furthermore, Koşar et al. (2007) compared microwave‐assisted hydrodistillation (MWHD) and HD for extracting EO from *F. vulgare* fruit and found that MWHD resulted in a 30% higher yield compared to HD. In our study, the EO yield attained through superheated steam distillation (SHSD) was the highest reported to date for *F. vulgare* seeds.

Overall, the results of our research indicate that the SHSD is a highly effective method for extracting essential oil (EO) from *F. vulgare* seeds, with a significantly higher yield compared to traditional hydrodistillation (HD) and steam distillation (SD) techniques. SHSD has also been shown to be the most effective technique for extracting EO from plant biomass, particularly at an industrial scale where it can save time and significantly increase yield. However, it should be acknowledged that numerous factors such as soil fertility, climate, harvesting and extraction seasons, and handling methods can also impact EO yield.

### 3.3 Antioxidant Activity of Essential Oils

DPPH‐FRSA, H_2_O_2_‐FRSA, and FRAP assays were used to conclude the antioxidant potential of *F. vulgare* Miller seeds EOs extracted by different extraction methods. The ability of essential oils to scavenge free radicals is greatly influenced by the method of extraction.

### 3.4 DPPH Free Radical Scavenging Activity

DPPH radical scavenging activity of essential oils refers to their capacity to neutralize DPPH radicals. DPPH is a synthetic organic compound that is used as a measure of a compound's antioxidant activity because it can accept an electron or hydrogen atom from a compound. It is often used to test the anti‐radical potential of phytochemicals because it is sensitive and requires minimal time for the experiment. The free radical scavenging compounds in essential oils react with DPPH, which turns yellow when it absorbs hydrogen from the antioxidant (Baliyan et al. 2022). The antioxidant potential is inversely proportional to the absorbance, so the absorbance was measured to estimate the antioxidant potential of EOs extracted using three methods: HD, SD, and SHSD. The current finding showed that EOs extracted using HD had the highest DPPH radical scavenging activity (75.52%), while those extracted using SHSD had the lowest activity (70%). The reason behind this could be the distinct chemical compositions of EOs obtained from these processes. It is important to mention that essential oils consist of volatile, aromatic compounds that possess diverse biological activities, like antimicrobial and antioxidant effects (Ayub et al. 2018). However, specific compounds and their proportions in an EO can differ reliant on the plant species and the extraction circumstances, meaning that different essential oils can have different biological activities. Some oils may have stronger antimicrobial effects but weaker antioxidant effects.

Our findings are in line with previous studies on DPPH‐FRSA of *F. vulgare* seeds essential oil mixed with sunflower oil, with Mazumder, Kumria, and Pathak (2014) reporting 76.84% FRSA. Shahat et al. (2011) also reported an IC_50_ value of 15.33 mg/mL FRSA for Iranian *F. vulgare* , while Faudale et al. (2008) found a range of 1230 to 411 μg/mL FRSA using the IC_50_ method on *F. vulgare* fruit essential oil from different Italian populations. Khammassi et al. (2018) observed FRSA IC_50_ values of 12–38 mg/mL for various *F. vulgare* Miller seed populations in Tunisia, and Anwar et al. (2009) reported an IC_50_ value of 32.32 μg/mL for DPPH FRSA.

### 3.5 H_2_O_2_ Scavenging Activity

H_2_O_2_ scavenging activity of essential oils refers to their ability to neutralize hydrogen peroxide, a compound that can cause damage to cells and tissues by acting as a pro‐oxidant in the body. H_2_O_2_ is a mild oxidant that can permeate cell membranes and react with Fe2+ and Cu2+ ions, resulting in the production of hydroxyl radicals, which can be detrimental to living organisms (Mazumder, Kumria, and Pathak 2014). It is therefore important to have compounds that can neutralize hydrogen peroxide and prevent it from causing harm.

Essential oils contain compounds that are able to scavenge hydrogen peroxide. In a study, the scavenging power of EOs extracted using the HD method was found to be the highest, at 68.25%. On the other hand, essential oils extracted using SHSD showed the minimum H_2_O_2_ scavenging activity, at 50.74%. When compared to ascorbic acid (a standard with 66.64% H_2_O_2_ scavenging activity), the HD extraction method yielded essential oils with enhanced H_2_O_2_ scavenging activity. However, essential oils extracted using the steam distillation (SD) method also showed good hydrogen peroxide scavenging activity. Evaluating the specific bioactive compounds in essential oils for their antioxidant activity can be challenging due to their complex compositions (Ayub et al. 2018). However, our results are consistent with previous research that found 71.61% H_2_O_2_ FRSA in the methanol extract of *F. vulgare* seeds (Goswami and Chatterjee 2014) and 70.04% H_2_O_2_ FRSA in the EO of *F. vulgare* seeds mixed with sunflower oil (Mazumder, Kumria, and Pathak 2014).

### 3.6 Ferric‐Reducing Ability (FRAP) Assay

The FRAP test is a method used to assess the antioxidant activity of phytocompounds. By measuring the change in absorbance at a specific wavelength, the antioxidant power of a substance is determined by its ability to reduce ferric ions to ferrous ions. FRAP examination is particularly useful for estimating the antioxidant activity of essential oils and can provide insight into their potential health benefits.

The principle behind this assay is to measure the number of electrons transferred from an antioxidant to Fe^3+^ by calculating the amount of Fe^2+^ produced. Concentration of ferrous ions is directly proportional to the ability of phytochemicals to donate electrons. Therefore, the FRAP value—a measure of the antioxidant potential of phytochemicals—is correlated with their ability to donate electrons (Chen et al. 2010). Gallic acid is used as the standard antioxidant in this assay, with the total antioxidant content of essential oils being expressed in terms of mg/L of gallic acid equivalent.

Total antioxidant content of essential oils obtained using various extraction techniques ranged from 113.72 to 210.32 mg/L GAE, as shown in Table 2. EO isolated using the superheated steam distillation (SHSD) technique exhibited the highest antioxidant content at 210.32 mg/L GAE, while the oil extracted using the hydrodistillation (HD) method had the lowest total antioxidant content at 113.72 mg/L GAE. Various extraction processes may separate different chemical components, which may account for the variability in antioxidant activity of essential oils.

**TABLE 2:** DPPH radical scavenging, H_2_O_2_ scavenging, as well as total antioxidant/FRAP activities of *F. vulgare* Miller seeds essential oils.

Distillation methods	DPPH FRSA (%)	Total antioxidant contents/FRPA (mg/100 g)	H_2_O_2_ FRSA (%)
Hydrodistillation	75.52 ± 0.05^b^	113.72 ± 0.96^c^	55.58 ± 0.09^b^
Steam distillation	73.68 ± 0.07^c^	188.35 ± 2.36^b^	44.39 ± 0.06^c^
Superheated steam distillation	70.00 ± 0.01^d^	210.32 ± 1.42^a^	38.60 ± 0.07^d^
Ascorbic acid	—	—	66.64 ± 0.08^a^
Gallic acid	81.03 ± 0.01^a^	—	—

*Note:* Values are mean ± Standard Deviations of three separate determinations. Different letters in superscript represent a significant difference among *F. vulgare* Miller seed essential oils extracted using different distillation methods. Antioxidant content/FRAP (measured as gallic acid equivalent, mg/L of essential oil).

A literature review found that the reducing power of *F. vulgare* Miller essential oil (EO) had not previously been measured using our methods. Therefore, we compared our outcomes to those found using similar methods in related studies. Marín et al. (2016) evaluated the reducing power of *F. vulgare* Miller EO at various concentrations (5, 10, 20, 50 g/L) and found reducing power values of 0.19, 0.26, 0.31, and 0.37, respectively, expressed as Trolox equivalent antioxidant capacity (TEAC) (Marín et al. 2016). Furthermore, Marín et al. (2016) determined the ferric‐reducing power of extracted material from *F. vulgare* Miller seeds by methanol extraction to be 1172.97 ± 0.005 𝜇mol/L.

These findings support the outcomes of our research, which revealed that the EOs extracted using the superheated steam distillation (SHSD) method had the highest antioxidant potential, while the EO extracted using the HD method had the lowest antioxidant potential, as determined using the same methods. Depending on the plant species and the conditions under which the oil is produced, the specific bioactive compounds and their proportions in an EO can differ. Different EOs can have different biological activities, with some exhibiting stronger antimicrobial effects and others having weaker antioxidant effects. However, current results propose that EOs from all distillation methods have significant antioxidant potential and can serve as a natural source of free radical scavengers, potentially providing various health benefits.

### 3.7 Antimicrobial Activity

Antimicrobial properties of EOs are well documented, with their ability to inhibit or kill various microorganisms such as bacteria, fungi, and viruses being widely recognized. In current research, the antimicrobial activity of *F. vulgare* seed EOs extracted through different methods was evaluated against bacterial and fungal species. The antibacterial activity was verified against *B. subtilis* , *S. aureus* , *P. multocida* , and *E. coli* , while the antifungal activity was assessed against *F. solani*, *A. niger* , A. *alternate*, and *A. flavus* . Inhibition zone diameters (mm) of EOs against bacterial and fungal strains were determined using disk diffusion. The minimum inhibitory concentrations (MICs) of all the EOs against all bacterial and fungal strains were determined using microdilution broth susceptibility assays and resazurin microtiter plate assays, respectively. Bacterial strains were assessed based on their percentage inhibition activity against biofilms.

The lipophilic nature of EOs allows to penetrate the microbial cell membrane, disrupting vital cellular activities and ultimately causing cell death. This occurs through the interaction of hydrophobic compounds in EOs with lipids in the plasma membrane and mitochondria, leading to the rupture of the cell membrane and the release of intracellular contents. It is worth noting that there are several target sites on microbial cells that can be affected by compounds with specific structures, ultimately impairing their vital functions.

The lipophilic nature of EOs permits them to easily penetrate the cell membranes of microbes and disrupt their vital activities (Calo et al. 2015). Specific structures of certain compounds originated in EOs can attach to specific target sites on microbial cells, effectively inhibiting their functions and leading to cell death (Ilić et al. 2019). Through their interaction with lipids in the cell membrane and mitochondria, these hydrophobic compounds disrupt the membrane's integrity, leading to the release of intracellular contents and ultimately causing cell death (Bajpai, Baek, and Kang 2012; Ju et al. 2019).

### 3.8 MIC and DIZ

The MIC and DIZ values of essential oils are useful indicators of their antimicrobial activity. In this study, the MIC value represents the lowest concentration of essential oil capable of inhibiting microorganism growth, while the DIZ value indicates the diameter of the zone of inhibition around a disk of essential oil placed on the culture. These values provide insight into the potential of EOs as natural antimicrobials and can be used to identify potential natural medicine candidates. In all test cases, the EOs demonstrated good antifungal activity against all fungi. The EO extracted from *F. vulgare* seeds using HD, SD, and SHSD methods showed DIZ values ranging from 10.81 to 18.78 mm and MIC values ranging from 0.31 to 10.0 μg against all tested fungal strains (Tables 3 and 4). In all tests, EO extracted using SHSD and SD methods was most effective, while EO extracted using HD exhibited the lowest antifungal potential against all tested strains. *Alternaria alternate* was the most sensitive strain against EO extracted using SD and SHSD, while *Aspergillus niger* was the least affected strain against EO extracted using HD (Tables 3 and 4). All strains showed less antifungal potential than the positive controls.

**TABLE 3:** Resazurin microtiter‐plate assay of *Foeniculum vulgare seed* essential oils isolated through different distillation methods.

Minimum inhibitory concentration (μg/mL)				
Fungal strains	Hydrodistilled EO	Steam‐distilled EO	Superheated steam‐distilled EO	Positive control*
*Fusarium solani* ATCC 36031	2.50 ± 0.06^a^	1.25 ± 0.05^b^	1.25 ± 0.03^b^	0.16 ± 0.02^c^
*Aspergillus niger* ATCC 10575	10.00 ± 0.05^a^	5.00 ± 0.02^b^	5.00 ± 0.03^b^	0.63 ± 0.00^c^
*Alternaria alternate* ATCC 20084	1.25 ± 0.02^a^	0.63 ± 0.04^b^	0.31 ± 0.02^b^	0.02 ± 0.01^c^
*Aspergillus flavus* ATCC 20046	5.00 ± 0.03^a^	2.50 ± 0.03^b^	2.50 ± 0.04^b^	0.32 ± 0.00^c^

*Note:* Triplicates Means ± Standard Deviations. Different letters in superscript represent a significant difference among *Foeniculum vulgare* Miller seed essential oils extracted by different distillation methods.

* Positive control for fungi was Fluconazole (25 μg/disc).

**TABLE 4:** Disk diffusion assay of *Foeniculum vulgare seed* essential oils isolated through different distillation methods.

Inhibition zone (mm)				
Fungal strains	Hydrodistilled EO	Steam‐distilled EO	Superheated steam‐distilled EO	Positive control*
*Fusarium solani* ATCC 36031	12.51 ± 0.03^d^	13.98 ± 0.06^c^	15.06 ± 0.08^b^	34.96 ± 0.18^a^
*Aspergillus niger* ATCC 10575	10.81 ± 0.06^d^	12.47 ± 0.04^c^	13.58 ± 0.06^b^	33.42 ± 0.28^a^
*Alternaria alternate* ATCC 20084	15.81 ± 0.04^d^	17.68 ± 0.07^c^	18.78 ± 0.04^b^	38.92 ± 0.15^a^
*Aspergillus flavus* ATCC 20046	11.82 ± 0.06^d^	13.78 ± 0.08^c^	14.78 ± 0.03^b^	34.52 ± 0.16^a^

*Note:* Triplicates Means ± Standard Deviations. Different letters in superscript represent a significant difference among *Foeniculum vulgare* Miller seed essential oils extracted by different distillation methods.

* Positive control for fungi was Fluconazole (25 μg/disc).

Our consequences are supported by various studies reporting the same range of MIC and DIZ values for *F. vulgare* EO against fungal strains. Similarly, AbduRahim et al. (2017) found out that *F. vulgare* seeds EO showed 0.125 μg/mL MIC value against *Aspergillus niger and Aspergillus flavus*. Khammassi et al. (2018) examined the antifungal activity of *F. vulgare* seeds EOs from different localities of Tunisia and reported MIC and DIZ results in a range of 2.5–7.5 μg/mL and 12.66–18.66 mm, respectively. Chang, Mohammadi Nafchi, and Karim (2016) reported 15.6–19.6 mm DIZ values of EO extracted from three Iranian *F. vulgare* seed varieties against *Aspergillus niger* (Chang, Mohammadi Nafchi, and Karim 2016). Ruberto et al. (2000) determined the antifungal activity of *F. vulgare* seeds aqueous extract against *Alternaria alternate* and *Aspergillus flavus* species and reported that *F. vulgare* seeds aqueous extract showed good activity against *Alternaria alternate* with an inhibition zone value of 32.3 mm. Similarly, Anwar et al. (2009) examined the antifungal activity of *F. vulgare* seed oil by disc diffusion and MIC assays from Pakistan flora. It was reported that *F. vulgare* seed EO revealed excellent antifungal activity against *Aspergillus niger and Fusarium solani*, with inhibition zones of 28 mm to 26 mm and MIC values of 80.6–91.1 mg/mL, respectively. Belabdelli et al. (2020) reported MIC values (0.2–0.25 μg/mL) and inhibition zone values (15–12 mm) of *F. vulgare* seeds EO against *Aspergillus flavus and Aspergillus nigger*. Barkat and Bouguerra (2012) found inhibition zone values of 20.67 and 21.67 mm of *F. vulgare* EO against *Aspergillus niger and Fusarium solani*, respectively.

The outcomes of the antimicrobial assays indicated that EO extracted using the SHSD method exhibited higher antibacterial and antifungal activity compared to the HD and SD methods. In EO, trans‐anethole, fenchone, limonene, and estragole were reported as the most prominent components in this study. The higher concentrations of these compounds may have contributed to the antimicrobial activity observed. Previous literature has also identified *trans*‐Anethole as a key contributor to the antimicrobial properties of *F. vulgare* Miller seeds essential oil (Cetin et al. 2010; Foroughi et al. 2017; Kazemi, Mousavi, and Kharestani 2012; Shahat et al. 2011). Additionally, previous research by Ilić et al. (2019) indicated that the antimicrobial action of *F. vulgare* fruit EOs is on the basis of the synergistic effect of the major compounds. However, Milenković et al. (2022) found it difficult to pinpoint a specific compound within the *F. vulgare* seeds essential oil that was responsible for a particular behavior.

It was concluded that the SHSD method was the most appropriate technique for the extraction of essential oils from *F. vulgare* seeds and could be used to produce natural antimicrobial compounds on a large scale. Further research is needed to fully understand the mechanisms behind the antimicrobial activity of these EOs and to determine the optimal concentrations and combinations of compounds for maximum efficacy. Antimicrobial activity of *F. vulgare* seed EOs extracted through different methods was evaluated against bacterial strains. A disk diffusion method was used to determine the diameter of the inhibition zone (DIZ), while the minimum inhibitory concentration (MIC) was determined through a micro‐dilution broth susceptibility assay. Current outcomes showed a range of DIZ values from 14.50 to 24.52 mm and a range of MIC values from 0.31 to 10.0 μg/mL for the *F. vulgare* seed EOs against bacterial strains (Tables 5 and 6). EOs extracted by superheated steam distillation (SHSD) displayed the highest antibacterial potential against all bacterial strains, while the EOs extracted by hydrodistillation (HD) demonstrated the lowest antibacterial potential. SHSD‐extracted EO also showed the highest DIZ value (24.52 mm) against *Pasteurella multocida* , while the lowest DIZ value (14.50 mm) was observed for HD‐extracted EO against *Staphylococcus aureus* . A similar pattern was observed in the MIC results, with SHSD‐extracted EO demonstrating the lowest MIC value (0.31 μg/mL) against *Pasteurella multocida* and the highest MIC (10.0 μg/mL) against *Escherichia coli* for HD‐extracted EO. Tables 5 and 6 show that the *F. vulgare* seed EOs extracted by HD, steam distillation (SD), and SHSD displayed lower MIC and DIZ values for *S. aureus* , *E. coli* , *B. subtilis* , and *P. multocida* compared to the positive control.

**TABLE 5:** Broth microdilution assay of *Foeniculum vulgare seed* essential oils isolated through different distillation methods.

Minimum inhibitory concentration (μg/mL)				
Bacterial strains	Hydrodistilled EO	Steam‐distilled EO	Superheated steam‐distilled EO	Positive control*
*Staphylococcus aureus* ATCC 25923	2.50 ± 0.04^a^	1.25 ± 0.03^b^	1.25 ± 0.02^b^	0.078 ± 0.02^c^
*Escherichia coli* ATCC 25922	10.00 ± 0.05^a^	5.00 ± 0.02^b^	5.00 ± 0.02^b^	0.31 ± 0.00^c^
*Bacillus subtilis* ATCC 10707	1.25 ± 0.02^a^	0.65 ± 0.02^b^	0.65 ± 0.01^b^	0.039 ± 0.02^c^
*Pastrulla multocida* ATCC 43137	0.63 ± 0.04^a^	0.31 ± 0.04^b^	0.31 ± 0.02^b^	0.01 ± 0.00^c^

*Note:* Triplicates Means ± Standard Deviations. Different letters in superscript represent a significant difference among *F. vulgare Miller* seed essential oils extracted by different distillation methods.

* Positive control for bacteria was Amoxicillin (25 μg/disc).

**TABLE 6:** Disk diffusion assay of *Foeniculum vulgare seed* essential oils isolated through different distillation methods.

Inhibition zone (mm)				
Bacterial strains	Hydrodistilled EO	Steam‐distilled EO	Superheated steam‐distilled EO	Positive control*
*Staphylococcus aureus* ATCC 25923	14.50 ± 0.11^c^	15.01 ± 0.12^b^	15.00 ± 0.15^b^	33.56 ± 0.18^a^
*Escherichia coli* ATCC 25922	15.51 ± 0.06^d^	17.50 ± 0.04^c^	18.00 ± 0.06^b^	38.00 ± 0.28^a^
*Bacillus subtilis* ATCC 10707	16.75 ± 0.13^c^	17.26 ± 0.14^b^	17.01 ± 0.16^b^	35.52 ± 0.14^a^
*Pastrulla multocida* ATCC 43137	22.01 ± 0.12^d^	24.01 ± 0.14^c^	24.52 ± 0.18^b^	39.12 ± 0.28^a^

*Note:* Triplicates Means ± Standard Deviations. Different letters in superscript represent a significant difference among *F. vulgare Miller* seed essential oils extracted by different distillation methods.

* Positive control for bacteria was Amoxicillin (25 μg/disc).

There have been various studies that have examined the antimicrobial activity of *F. vulgare* Miller seeds essential oil (EO) (AbduRahim et al. 2017; Diao et al. 2014; Ghasemian et al. 2020; Upadhyay 2015). These studies have consistently shown that the EO is generally less effective against *S. aureus* than it is against *E. coli* . AbduRahim et al. (2017) found that the EO exhibited a DIZ of 19 mm and 20 mm for *S. aureus* and *E. coli* , respectively, and had a MIC of 0.781 μL/mL for both *S. aureus* and *E. coli* . Diao et al. (2014) also observed that the EO had a lower DIZ of 11.50 mm for *S. aureus* compared to 19.10 mm for *E. coli* . Ghasemian et al. (2020) reported DIZ values of 18 mm and 20 mm for *S. aureus* and *E. coli* , respectively, while Upadhyay (2015) found DIZ values of 21.60 mm and 24.63 mm for *S. aureus* and *E. coli* , respectively, and MIC values of 48 and 6.0 μg/mL, respectively. Abdellaoui, Derouich, and El‐Rhaffari (2020) reported DIZ values of 18.35, 12.84, and 13.26 mm, and MIC values of 125, 250, and 250 μg/mL for *E. coli* , *S. aureus* , and *B. subtilis* , respectively. Khammassi et al. (2018) examined EOs from different localities in Tunisia and found a range of MIC values (2.5–7.5, 1.5–7.5, 5–7.5 μg/mL) and DIZ values (10.33–14.33, 11.66–15.66, 9.33–12.66 mm) against *E. coli* , *S. aureus* , and *B. subtilis* , respectively. Chang, Mohammadi Nafchi, and Karim (2016) reported DIZ values of 9.5–15.5 mm, 13.8–18.7 mm, and 12.6–15.5 mm for *E. coli* , *S. aureus* , and *B. subtilis* , respectively, for three Iranian varieties of *F. vulgare* Miller seed EOs. Mazumder, Kumria, and Pathak (2014) found MIC values of 3, 4, and 4 μg/mL against *E. coli* , *S. aureus* , and *B. subtilis* , respectively. Ruberto et al. (2000) reported DIZ values of 7.3 mm, 10.7 mm, and 9.0 mm for *E. coli* , *S. aureus* , and *B. subtilis* , respectively, for the aqueous extract of *F. vulgare* seeds. Anwar et al. (2009) found DIZ values of 14 mm and 29 mm, and MIC values of 249.3 and 62.6 mg/mL for *E. coli* and *B. subtilis* , respectively. Ilić et al. (2019) reported DIZ values of 18, 19, and 28 mm, and an MIC value of 100, 75, and 25 μg/mL for *E. coli* , *S. aureus* , and *B. subtilis* , respectively, for the integrated *F. vulgare* fruit EO. Finally, Amat et al. (2019) reported a minimum inhibitory concentration (MIC) value of 0.013% V/V for *F. vulgare* EO against *Pastrulla multocida*.

Depending on the extraction method and the specific bacterial or fungal strain, *F. vulgare* seed EOs may have different levels of activity. *F. vulgare* seed EOs can be used as antimicrobial agents, but further research is needed to fully understand their mechanisms of action.

### 3.9 Biofilm Inhibition Activity

The biofilm inhibition activity of essential oils refers to their ability to prevent the formation or disrupt the existing biofilms, which are microorganisms that adhere to surfaces and create a defending layer. These biofilms, consisting of bacteria, fungi, and viruses, can be challenging to remove and may cause health issues. EOs have been demonstrated to show biofilm inhibition activity, meaning that they can prevent the formation or disrupt existing biofilms. The biofilm inhibition activity of essential oils is a crucial property that contributes to their potential health benefits. It has the potential to hinder the growth of harmful biofilms on surfaces and within the body.

During biofilm formation, essential oils demonstrate antibacterial potential against both sessile and planktonic cells. They enhance bacterial cell membrane penetrability, leading to the disruption of cell membrane integrity and the inhibition of bacterial growth (Rossi et al. 2022). Among the different extraction methods, EOs extracted by SHSD exhibited the highest antibacterial potential against biofilms of all bacterial strains, while the EOs extracted by HD exhibited the lowest antibacterial potential against biofilm bacterial strains. EOs of *F. vulgare* seeds presented a range of 24.95%–49.46% inhibition activity against biofilms of all tested bacterial strains. Table 7 demonstrates that the EOs isolated by SHSD exhibited the highest inhibition activity (49.46%) against *Pastrulla multocida*, while the lowest inhibition activity (24.95%) was shown by EO extracted by HD against *Staphylococcus aureus* biofilm. Our research indicates that there is no available literature to compare our findings on % inhibition activity against the specific bacterial strains of interest.

**TABLE 7:** Biofilm inhibition activity of *Foeniculum vulgare seed* essential oils isolated through different distillation methods.

Biofilm inhibition activity (%)			
Bacterial strains	Hydrodistilled EO	Steam‐distilled EO	Superheated steam‐distilled EO
*Staphylococcus aureus* ATCC 25923	24.95 ± 0.04^c^	26.40 ± 0.06^b^	30.92 ± 0.08^a^
*Escherichia coli* ATCC 25922	29.11 ± 0.07^c^	32.01 ± 0.06^b^	34.01 ± 0.09^a^
*Bacillus subtilis* ATCC 10707	29.65 ± 0.06^c^	31.24 ± 0.08^b^	36.04 ± 0.09^a^
*Pastrulla multocida* ATCC 43137	44.34 ± 0.05^c^	47.23 ± 0.04^b^	49.46 ± 0.06^a^

*Note:* Triplicates Means ± Standard Deviations. Different letters in superscript represent a significant difference among *Foeniculum vulgare* Miller seed essential oils extracted by different distillation methods.

## 4 Conclusion

This study demonstrates the potential of superheated steam distillation (SHSD) as a highly efficient and effective method for extracting high‐quality essential oils from *Foeniculum vulgare* Miller seeds. The SHSD method yielded a higher concentration of major compounds, namely trans‐anethole, estragole, fenchone, and limonene, compared to the conventional hydrodistillation and steam‐distillation methods. Moreover, essential oil extracted by SHSD showed superior antioxidant, antibacterial, and antifungal activities compared to those obtained by the other two methods. The results of this study suggest that SHSD can be a promising alternative procedure for extracting EO from *F. vulgare* seeds, which may find applications in the fields of cosmetics, pharmaceuticals, and food. However, optimization of SHSD conditions, including temperature, particle size, extraction time, and steam flow rate, is essential to further enhance the yield and quality of the EOs. Thus, future studies should focus on fine‐tuning these parameters to maximize the yield of EOs and to improve the efficiency of the SHSD method. As a result of this study, SHSD can be used as an alternative method for extracting essential oils from medicinal plants that offers unique advantages. These insights could lead to increased export of EOs from *F. vulgare* seeds and other valuable medicinal plants, which would benefit the food, cosmetic, and medicinal industries.

## Author Contributions

**Muhammad Haseeb Raza:** conceptualization (equal), methodology (equal), writing – original draft (equal). **Muhammad Adnan Ayub:** formal analysis (equal), supervision (equal), writing – original draft (equal). **Muhammad Zubair:** writing – review and editing (equal). **Amjad Hussain:** data curation (equal), methodology (equal), writing – review and editing (equal). **Samreen Saleem:** formal analysis (equal), validation (equal). **Muhammad Tauseef Azam:** data curation (equal), investigation (equal), methodology (equal). **Muzzamal Hussain:** writing – review and editing (equal). **Anjuman Gul Memon:** conceptualization (equal), writing – review and editing (equal). **Mohamed A. Abdelgawad:** investigation (equal), visualization (equal). **Mohammed M. Ghoneim:** formal analysis (equal), visualization (equal). **Ahmed H. El‐Ghorab:** project administration (equal), resources (equal), validation (equal). **Ehab M. Mostafa:** data curation (equal), software (equal). **Entessar Al Jbawi:** data curation (equal), supervision (equal).

## Conflicts of Interest

The authors declare no conflicts of interest.

## Data Availability

The data that support the findings of this study are available on request from the corresponding author.
